# The Hardest Mode to Die

**Published:** 1854-07

**Authors:** 


### THE HARDEST MODE TO DIE.

To be shot dead is one of the easiest modes of terminating life; yet, rapid as it is, the body has leisure to feel and reflect. On the first attempt by one of the frantic adherents of Spain, to assassinate William, Prince of Orange, who took the lead in the revolt of the Netherlands, the ball passed through the bones of the face and brought him to the ground. In the instant that preceded stupefaction, he was able to frame the notion that the ceiling of the room had fallen and crushed him.

The cannon shot which plunged into the brain of Charles XII., did not prevent him from seizing his sword by the hilt. The idea of an attack, and the necessity for defence, was pressed on him by a blow which we should have supposed too tremendous to leave an interval for thought. But it by no means follows that the inflicting of fatal violence is accomplished by a pang. From what is known of the first effect of gun-shot wounds, it is probable that the impression is rather stunning than acute. Unless death be immediate, the pain is as varied as the nature of the injuries, and these are past counting up.

But there is nothing singular in the dying sensation, though Lord Byron remarked the physiological peculiarity that the expression is invariably that of languor; while in death from a stab, the countenance reflects the traits of natural character, of gentleness or ferocity, to the last breath.

Some of these cases are of interest to show with what slight disturbance life may go under a mortal wound, till it finally comes to a sudden stop. A foot soldier at Waterloo, pierced by a musket ball in the hip, begged water of a trooper, who chanced to possess a canteen of beer. The wounded man drank, returned his heartiest thanks, mentioned that his regiment was nearly exterminated, and having proceeded a dozen yards on his way to the rear, fell to the earth, and with one convulsive movement of his limbs, concluded his career. "Yet his voice," says the trooper, who himself tells the story, "gave scarcely the smallest sign of weakness."

Captain Basil Hall, who, in his early youth, was present at the battle of Corunna, has singled out, from the confusion which consigns to oblivion the woes and gallantry of war, anothei instance, extremely similar, which occurred on that occasion. An old officer, who was shot in the head, arrived pale and faint at the temporary hospital, and begged the surgeon to look at his wound, which was pronounced mortal. "Indeed, I feared so," he responded, with impeded utterance, "and yet I should like to live a little longer were it possible." He laid his sword upon a stone, at his side, "as gently," says Hall, "as if its steel had been turned to glass, and almost immediately sank dead upon the turf."—*Quarterly Review.*

